# Using polygenic risk modification to improve breast cancer prevention: study protocol for the PRiMo multicentre randomised controlled trial

**DOI:** 10.1136/bmjopen-2024-087874

**Published:** 2024-08-05

**Authors:** Simone McInerny, Lyon Mascarenhas, Tatiane Yanes, Lara Petelin, Georgia Chenevix-Trench, Melissa C Southey, Mary-Anne Young, Paul A James

**Affiliations:** 1 Parkville Familial Cancer Centre, Peter MacCallum Cancer Centre, Melbourne, Victoria, Australia; 2 Parkville Familial Cancer Centre, The Royal Melbourne Hospital, Parkville, Victoria, Australia; 3 Frazer Institute, Dermatology Research Centre, The University of Queensland, Brisbane, Queensland, Australia; 4 The Daffodil Centre, joint venture with Cancer Council NSW, The University of Sydney, Sydney, New South Wales, Australia; 5 The University of Melbourne School of Population and Global Health, Melbourne, Victoria, Australia; 6 Cancer Genetics Laboratory, QIMR Berghofer Medical Research Institute, Herston, Queensland, Australia; 7 Precision Medicine, Monash University School of Clinical Sciences at Monash Health, Clayton, Victoria, Australia; 8 Cancer Council Victoria Cancer Epidemiology Division, Melbourne, Victoria, Australia; 9 Clinical Translation and Engagement Platform, Garvan Institute of Medical Research, Darlinghurst, New South Wales, Australia; 10 School of Clinical Medicine, St Vincent's Healthcare Clinical Campus, Faculty of Medicine and Health, UNSW Sydney, Sydney, New South Wales, Australia; 11 Sir Peter MacCallum Department of Oncology, The University of Melbourne, Melbourne, Victoria, Australia

**Keywords:** Cancer genetics, Breast tumours, Clinical Trial

## Abstract

**ABSTRACT:**

**Introduction:**

Established personal and familial risk factors contribute collectively to a woman’s risk of breast or ovarian cancer. Existing clinical services offer genetic testing for pathogenic variants in high-risk genes to investigate these risks but recent information on the role of common genomic variants, in the form of a Polygenic Risk Score (PRS), has provided the potential to further personalise breast and ovarian cancer risk assessment. Data from cohort studies support the potential of an integrated risk assessment to improve targeted risk management but experience of this approach in clinical practice is limited.

**Methods and analysis:**

The polygenic risk modification trial is an Australian multicentre prospective randomised controlled trial of integrated risk assessment including personal and family risk factors with inclusion of breast and ovarian PRS vs standard care. The study will enrol women, unaffected by cancer, undergoing predictive testing at a familial cancer clinic for a pathogenic variant in a known breast cancer (BC) or ovarian cancer (OC) predisposition gene (*BRCA1*, *BRCA2*, *PALB2*, *CHEK2*, *ATM*, *RAD51C*, *RAD51D*). Array-based genotyping will be used to generate breast cancer (313 SNP) and ovarian cancer (36 SNP) PRS. A suite of materials has been developed for the trial including an online portal for patient consent and questionnaires, and a clinician education programme to train healthcare providers in the use of integrated risk assessment. Long-term follow-up will evaluate differences in the assessed risk and management advice, patient risk management intentions and adherence, patient-reported experience and outcomes, and the health service implications of personalised risk assessment.

**Ethics and dissemination:**

This study has been approved by the Human Research Ethics Committee of Peter MacCallum Cancer Centre and at all participating centres. Study findings will be disseminated via peer-reviewed publications and conference presentations, and directly to participants.

**Trial registration number:**

ACTRN12621000009819.

**Strengths and limitations of this study**

- The trial is an Australian nationwide, multicentre randomised controlled trial that will generate high-level evidence on the clinical, psychosocial and health implications of integrated breast and ovarian cancer risk assessment in clinical practice.
- Participants will be enrolled following referral for clinical predictive genetic testing, allowing study activities to be integrated with existing clinical processes, and include both individuals who test positive and negative for a pathogenic variant.
- A limitation is that the breast and ovarian cancer Polygenic Risk Score employed in the trial were developed in European populations and are expected to be less predictive for many women in the ancestrally diverse Australian population, requiring additional adjustment to account for this effect.

## Introduction

 The hereditary component underlying breast and ovarian cancer risk is one of the highest for any solid tumour, meaning that women at highest risk can be identified through genetic testing combined with personal and family risk factors, using validated risk assessment tools.^1 2^ A highly effective way to prevent breast cancer deaths is to target risk management strategies to those at higher risk. At the high end of familial risk, genetic testing is well-established for rare pathogenic variants (PVs) in a panel of breast cancer genes.^3^ The best known are *BRCA1* and *BRCA2* which convey average lifetime breast cancer risks of 50% to 70% and significant risks for ovarian cancer and other malignancies.^4^

For women who carry a PV in *BRCA1* or *BRCA2,* evidence shows that risk management options, including screening from a young age, breast MRI screening, risk-reducing medication and prophylactic mastectomy and oophorectomy, are effective.^5^ The current panel of genes with strong evidence for use in clinical practice include *PALB2, CHEK2*, *ATM*, *RAD51C* and *RAD51D* with additional genes such as *BARD1* (breast cancer) and *BRIP1* (ovarian cancer) continuing to emerge. Notably, with the exception of *PALB2*,^6^ the breast cancer risk for these more recently described genes is substantially lower than for *BRCA1* and *BRCA2* and they are commonly described as moderate risk genes (risks twofold to fourfold compared with the population). The clinical model developed over years to manage the clear high risks of *BRCA1* and *BRCA2* has had to adapt to the complexity introduced by greater clinical uncertainty from both the less mature evidence and the more moderate risks associated with the recently identified genes. However, genetic testing, including all of these genes, is now a standard part of breast cancer care.^7^

In parallel initiatives, hundreds of common single nucleotide variations or polymorphisms (SNPs) in the genome have been identified from large genome-wide association studies, each of which is associated with a small risk of breast cancer.^8^,^10^ When combined into a single measure, known as a Polygenic Risk Score (PRS), this information can be used to describe the distribution of breast cancer risk in the general population^8^ and in familial breast cancer.^11^ In contrast to rare variants, PRS can be applied to all women. The magnitude of risk for different levels of polygenic risk has been established in large studies^12^ and validated prospectively.^13^

Initial studies that examined the effect of a PRS on breast cancer risk in *BRCA1* and *BRCA2* carriers showed the absolute lifetime risk of breast cancer was around 10%–20% greater for carriers in the highest decile of the PRS distribution compared with the lowest.^14^ These effects have been further examined for breast and ovarian cancer with expanded PRS: 313 SNPs for breast^8^ and 36 SNPs for ovarian cancer.^15^ Potentially, even more significant variation has been demonstrated with the combination of the PRS and moderate-risk genes such as *CHEK2*. A study of 844 carriers of a *CHEK2* PV found an average lifetime breast cancer risk close to the population level (14.3%) in the 20% of carriers with the lowest PRS and a high risk (32.6% lifetime risk) in the same proportion of women with the highest PRS.^16^

The psychological outcomes of germline genetic testing for *BRCA1* and *BRCA2* are well understood. Studies show little evidence of negative impacts in the longer term in those carrying a PV, and some benefits for those found to be non-carriers.^17^,^20^ Women’s responses and understanding of polygenic breast cancer risk information (the PRS) are less well researched. A long-term Australian study (the ViP study) examining the role of the PRS in clinical risk assessment^11^ showed a high level of comprehension and acceptance of receiving such information^21^,^23^ and has informed the patient care model for the current trial.

Continuing concerns about the use of PRS information include its use in non-European populations.^24^ The most widely researched PRS was developed using data from women of European background and are both less well characterised and, where examined, are less predictive in women of all other ancestral backgrounds.^25^ In addition, there are concerns that the PRS effects are only moderate, requiring a different conceptual model for PRS-based risk assessment compared with single gene approaches. A recent review of the role of PRS in clinical care concluded that there is not yet unequivocal evidence of demonstrated clinical benefit in any setting^26^ and more clinical data is needed before PRS is ready for routine implementation for breast cancer risk assessment.^27^ Large well-designed randomised controlled trials (RCTs) are needed to assess outcomes such as the accuracy of PRS-informed risk assessments and their effect on risk-reducing strategies and breast cancer incidence, acceptability and understanding of PRS information, and the psychological and health system impacts.

Currently, thousands of women attend familial cancer clinics (FCCs) annually across Australia to be tested for a PV in a high-risk or moderate-risk gene found in their family as a cause for breast (±ovarian) cancer. These women represent an important potential study group for an initial examination of the clinical value of PRS risk modification as they are already undergoing predictive genetic testing, allowing the study to be integrated with existing clinical processes. Here, we describe the polygenic risk modification (PRiMo) trial, an Australian multicentre prospective randomised controlled trial of integrated risk assessment including personal and family risk factors with inclusion of breast and ovarian PRS vs standard care. The protocol is based on the Standard Protocol Items: Recommendations for Interventional Trials (SPIRIT) recommendations,^28^ and a completed SPIRIT checklist is available (see online supplemental file).

## Methods and analysis

### Aims

The primary aim of the trial is to compare the performance of an integrated assessment of breast and ovarian cancer risk that incorporates PRS risk modification, along with personal and familial risk factors, with the current standard of care in a cohort of Australian women undergoing predictive genetic testing for a breast/ovarian cancer gene at an FCC. In the short term, the study will compare the distribution of assessed risk, and risk management recommendations and intentions in the PRiMo intervention group vs the standard care group. In the medium to long term, the study will (i) assess uptake and adherence to risk management strategies and (ii) use follow-up of prospective cancer events to examine the accuracy and calibration of the risk assessment between the PRiMo risk assessment and standard care.

Secondary aims are to assess patient attitudes and experience when undergoing personalised genomic risk assessment for breast and ovarian cancer and to establish the health service implications of implementing PRS-risk-modified assessments in clinical practice including a health economic evaluation.

### Design and setting

The PRiMo intervention is being evaluated using a two-arm superiority multicentre RCT. Study participants will be recruited through 11 dedicated FCCs around Australia where unaffected women are referred for predictive genetic testing of high-risk or moderate-risk breast or ovarian cancer genes, following detection of a likely pathogenic (class 4) or pathogenic (class 5) variant in a family member. Participants in the RCT are allocated to either (i) a standard care group, who undergo predictive testing as per the FCC’s usual practice or (ii) an intervention group, where the genetic healthcare provider (GHP) at their FCC provides them with risk assessment and management advice based on the PRiMo risk assessment, which integrates their rare PV genetic test result, breast and ovarian cancer PRS, detailed family history information and personal risk factors. Based on consumer input the study design allows participants in the standard care group to crossover and receive their PRiMo risk assessment including the PRS, 1 year after their standard results appointment so that this is eventually available to all participants.

Although comparatively rare, PVs in breast cancer predisposition genes other than *BRCA1* and *BRCA2* are of particular interest given that in this context, the PRS results can have increased implications for risk stratification. To ensure adequate representation of these genes, the study will also recruit a retrospective cohort of women who have previously been found to carry a PV in one of these genes. These participants will be provided with updated risk management advice from a GHP at their FCC based on the PRiMo risk assessment.

**Figure 1:**
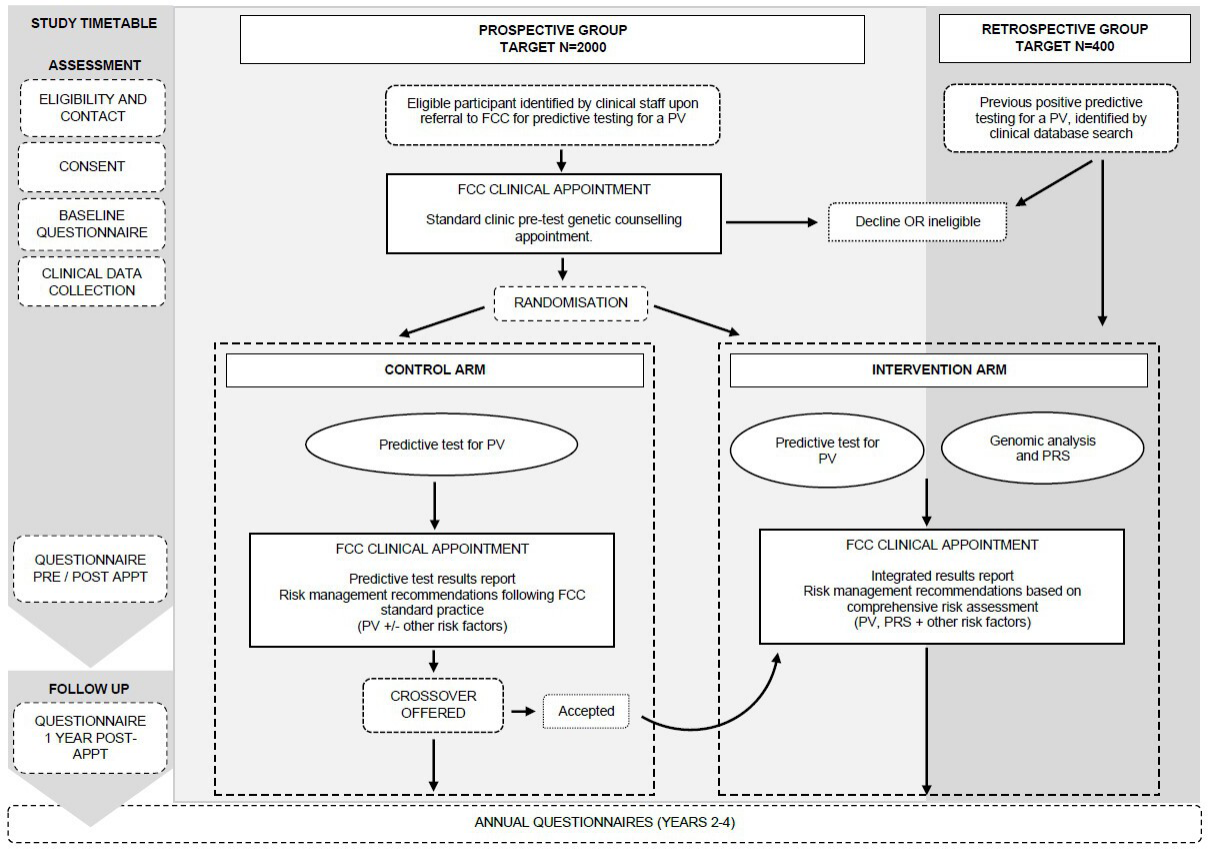
Study procedures flowchart. APPT, appointment; FCC, Familial Cancer Centre; PRS, Polygenic Risk Score; PV, pathogenic variant.

The study opened at the first site in July 2021 and is expected to take at least 5 years including recruitment and follow-up. Figure 1 summarises the study procedures for the prospective RCT and the retrospective cohort.

### Intervention

Standard care at participating FCCs currently provides risk assessment and risk management advice based on the result of the predictive gene test, along with basic risk factor information based on the national guidelines on cancer risk management for gene carriers.^29^ Where additional personal risk factor information is included, the risk is typically incorporated using the CanRisk tool—a validated online tool to calculate an integrated lifetime breast and ovarian cancer risk.^1 30^ This tool has been shown to be acceptable to clinicians in the genetics setting,^31^ although its use in clinical practice in Australian FCCs was variable at the time the trial commenced. The CanRisk tool is supported by grant PPRPGM-Nov20\100 002 from Cancer Research UK.

The PRiMo intervention is an enhanced breast cancer risk assessment that combines PRS calculated from a combination of common genomic variants with the result of a predictive genetic test and a standard set of basic risk factor information (age, lifestyle risk factors and family history of cancer). This personalised risk assessment is calculated using the CanRisk tool and the output summarised in a custom report that includes the primary genetic information and a risk summary of 5-year, 10-year and lifetime risks for breast and ovarian cancers. In both arms, risk management advice, ongoing follow-up and clinical care will be determined by the participant’s managing FCC and medical team. The retrospective cohort participants have previously tested positive for a moderate-risk gene and had a ‘standard care’ results appointment. Consenting participants in this group will all receive a PRiMo risk assessment and the analysis of the primary study aims will involve comparison of pre-result and post-result questionnaire data for the same women.

The use of PRS in an integrated individualised risk assessment is a novel element in clinical practice. To ensure that clinicians feel confident, and the study intervention is delivered in a standard manner, the trial included development of an education and training programme used with GHPs at participating sites. The programme comprises online learning covering the theoretical aspects of PRS and a facilitated virtual workshop with pre-recorded roleplays and case discussions.^32^

### Sample

#### Prospective RCT

The trial aims to recruit up to 2000 women undergoing a predictive genetic test for a known familial PV in a gene associated with increased risk of breast cancer: *BRCA1, BRCA2, PALB2, CHEK2, ATM, RAD51C* and *RAD51D*. Eligible participants are 18 to 79 years of age, unaffected by invasive or in situ breast cancer or epithelial ovarian cancer, and able to give informed consent and complete online questionnaires in English.

Exclusion criteria include having had previous genomic testing that included polygenic risk information for breast or ovarian cancer and currently undergoing treatment for a cancer diagnosis.

#### Retrospective cohort

To ensure adequate representation of the rarer genes, the trial is inviting up to 400 women who have previously tested positive for a PV in *PALB2, CHEK2, ATM*, *RAD51C* or *RAD51D*. Other inclusion and exclusion criteria are as for the prospective RCT.

### Recruitment

#### Prospective RCT

Potential participants are identified by the individual FCCs following referral for predictive testing for a PV detected in a genetic relative. The managing FCC directs eligible patients to the PRiMo online information and consent platform where they can complete the consent form prior to or within 1 day following the standard pre-test counselling appointment with the FCC where the GHP will confirm their willingness to participate and answer questions.

#### Retrospective cohort

Potential participants are identified from the databases of the FCCs and sent an invitation letter or email inviting them to access the PRiMo online information and consent platform. Individuals who complete the consent form are phoned by a GHP or researcher to verbally re-confirm their understanding and willingness to participate and to organise their FCC results appointment. Individuals not interested in the study are asked to return the decliner response form and non-responders may be followed up by the FCC.

#### Online information and consent platform

The PRiMo online information and consent platform uses REDCap (Research Electronic Data Capture) tools, a secure, web-based software platform designed to support data capture for research studies.^33 34^ On accessing the platform, potential participants are provided with information specific to the prospective and retrospective groups and then an online version of the detailed participant information sheet. Interested individuals provide basic information, complete screening questions to confirm eligibility and electronically sign the consent form. The consent form allows participants to ‘opt out’ of being invited to participate in the qualitative interviews and focus groups, and provides participants with the option to consent to their data and samples being included in other closely related future research projects that have been approved by a Human Research Ethics Committee. Following the pre-test counselling appointment or phone call, participants are provided with a separate online optional consent form that provides permission for the researchers to access their data from the Australian Government Medicare Benefits Schedule (MBS) and Pharmaceutical Benefits Scheme (PBS).

### Genomic analysis and PRS

The predictive test is performed by the accredited molecular diagnostic laboratory for each FCC independently of the study and the standard clinical test report is provided to the FCC and the central study team. An additional aliquot of the DNA sample is provided by the diagnostic laboratory for the research genomic analysis.

SNP genotyping is performed on a custom Illumina GSA array. Quality control and genotype calling of the array data are performed using standard bioinformatic packages. Missing genotypes, either due to limitations of the array or issues arising from individual samples, are managed by imputation against the TOPMED and 1000 Genomes whole-genome data sets using standard methodology to optimise the imputation accuracy for each SNP.

Calculation of a breast cancer PRS (BCAC-313) and ovarian cancer PRS (OCAC-36) and generation of the report are performed by the central study team using standard methods developed in the pilot study.^21 22^ Ancestry information is incorporated into the breast cancer risk prediction; information on self-reported ethnicity and published figures on the characteristics of the BCAC-313 PRS score specific to different ancestral groups^25 35 36^ to provide an adjusted Z-score for incorporation into the multifactorial assessment.

### Randomisation

Randomisation occurs following completion of the pre-test counselling appointment and pre-result baseline questionnaire. Participants in the prospective RCT are randomly assigned to either standard care or PRiMo intervention with a 1:1 allocation as per a computer-generated simple randomisation schedule using the REDCap randomisation module. The trial staff allocating participants do not have access to the randomisation schedule.

To minimise discrepancies in risk advice provided to relatives managed by the same FCC, only the first participant in a family at that site will be randomly assigned to either standard care or intervention. Any related participants that attend the same FCC will be allocated to the same group. Participants will not be informed of this procedure and potential participants will not be aware of their group prior to enrolment.

Given the nature of the intervention, participant and clinician blinding is not possible and both the participant and their FCC will be informed of the outcome following allocation, and procedures for unblinding are not required. Analysis of the primary study aims will be performed without knowledge of the intervention status of each group.

Participants in the retrospective cohort will not be randomised and all will receive the intervention.

### Study outcome measures and data collection

**Figure 2:**
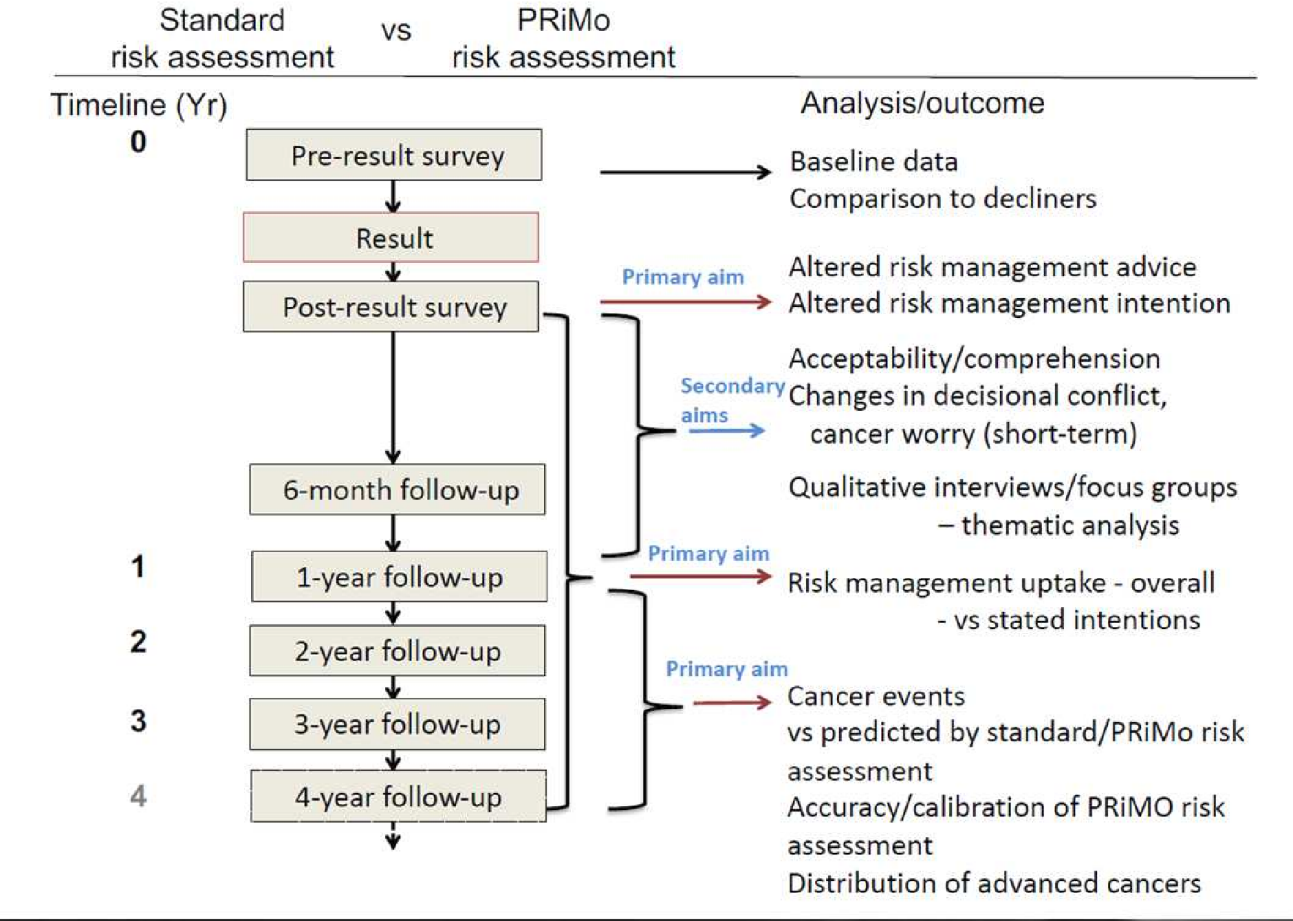
Timetable of data collection for primary and secondary study aims.

The data collection timetable is depicted in figure 2 showing the outcomes comparisons that will address the primary and secondary aims.

#### Quantitative data

Data will be collected before participants attend their FCC to receive their genetic test results, risk assessment and risk management advice (pre-result) and at set timepoints after their results, as outlined in table 1.

**Table 1:** Participant questionnaires and clinical data collection at each timepoint

	Timepoint						
	Pre-result	Post-result					
		1 month	6 months	1 year	Crossover*	2 years	3 years
Participant questionnaires							
Demographic data	X						
Risk factors: lifestyle, personal, reproductive, medical and medications	X			X		X	X
Risk management intentions		X			*X*		
Risk management uptake				X		X	X
Cancer events	X		X	X		X	X
Quality of life and psychosocial instruments:							
Assessment of Quality of Life (AQoL)−8D^40^	X	X	X	X	*X*		
Cancer risk perception^43^	X	X	X	X	*X*		
Cancer Worry Scale (CWS)^44^	X	X	X	X	*X*		
Genomics Outcome Scale (GOS)^45^	X	X			*X*		
List of Threatening Experiences (LTE)^46^	X	X	X	X	*X*		
Feelings About genomiC Testing Results (FACToR)^47^		X	X	X	*X*		
Post-traumatic Growth Index - Short Form (PGI-SF)^48^		X	X	X	*X*		
Psychological Adaptation to Genetic Information Scale (PAGIS)^49^		X	X	X	*X*		
Personal Utility of Genomic Results (PrU)^50^		X	X	X	*X*		
Clinical data collection							
Risk factors: family cancer history (pedigree) and PV predictive test report	X						
Risk assessment and risk management recommendations		X			*X*		
Medicare Benefits Schedule (MBS) and Pharmaceutical Benefits Scheme (PBS) claims (optional consent)							X

* One month post-second results appointment for standard care arm participant that has chosen to receive PRiMo intervention (crossover).

PVpathogenic variant

#### Qualitative data

Qualitative interviews (up to 60) complement and triangulate the psychosocial questionnaire data with the sample purposefully selected to cover a range of risk and demographic profiles. The first interview will occur 1 to 4 months after result disclosure and a second interview conducted at about 12 months. The recorded, semistructured interviews (45 to 60 min) will be conducted by a trained psychosocial researcher, covering the experience of receiving personalised risk information (motivation for genetic testing, expectations and understanding of information); risk perception and management strategies planned or undertaken; the impact of results and changes over time; and (dis)advantages of participation.

Semistructured focus groups will be conducted with approximately 5–30 participants (3 to 8 per group) to explore perspectives towards the future implementation of PRS for breast/ovarian cancer risk assessment in clinical genetics, including barriers and enablers. To achieve a representative sample, participants will be purposefully recruited to reflect the range of age, genetic result and cancer risk. Focus groups will be moderated by a trained researcher using a guide informed by two validated implementation determinant frameworks.^37 38^

### Statistical considerations and sample size

A power calculation was performed based on the frequency of cancer risk management events in cases and controls—including uptake of recommended strategies and reduction in strategies no longer recommended based on the participants’ risk assessment. Significance is set at p≤0.05 for independent direct comparisons of the two arms, and p≤0.01 after correction for multiple testing for comparisons between subgroups (gene positive; gene negative; and high, moderate and population risk). In the prospective enrolment group, the sample size has power to detect 10% differences in proportions between arms (power for χ^2^ test exceeds α≤0.05, β≥0.8 at 85% of enrolment of prospective group) and power to detect 25% differences in the smallest risk subgroup (moderate risk), accounting for multiple testing. Comparison of the prospective accuracy of PRiMo vs standard care risk assessments was based on a model of the expected distribution of genetic testing results and the PRS that projected an expectation of incident cancers in follow-up. At 3 years mean follow-up, the full sample will be powered to detect a 10% increase in the proportion of breast cancers detected in the high-risk group; that is, a greater proportion of the breast cancers detected in follow-up will occur in the high-risk women in the intervention arm than in the standard care arm (β=88%, α=0.05, on a test for superiority). The sample for retrospective recruitment was based on the number of families with an identified PV in *PALB2*, *ATM* or *CHEK2* in Australia.

For the secondary aims, the proposed study size correlates to more than adequate statistical power (β>90%, α=0.01) to detect differences in the questionnaire outcomes. Consideration will be given to both the statistical and clinical significance of any differences.

### Data analysis

**Figure 3:**
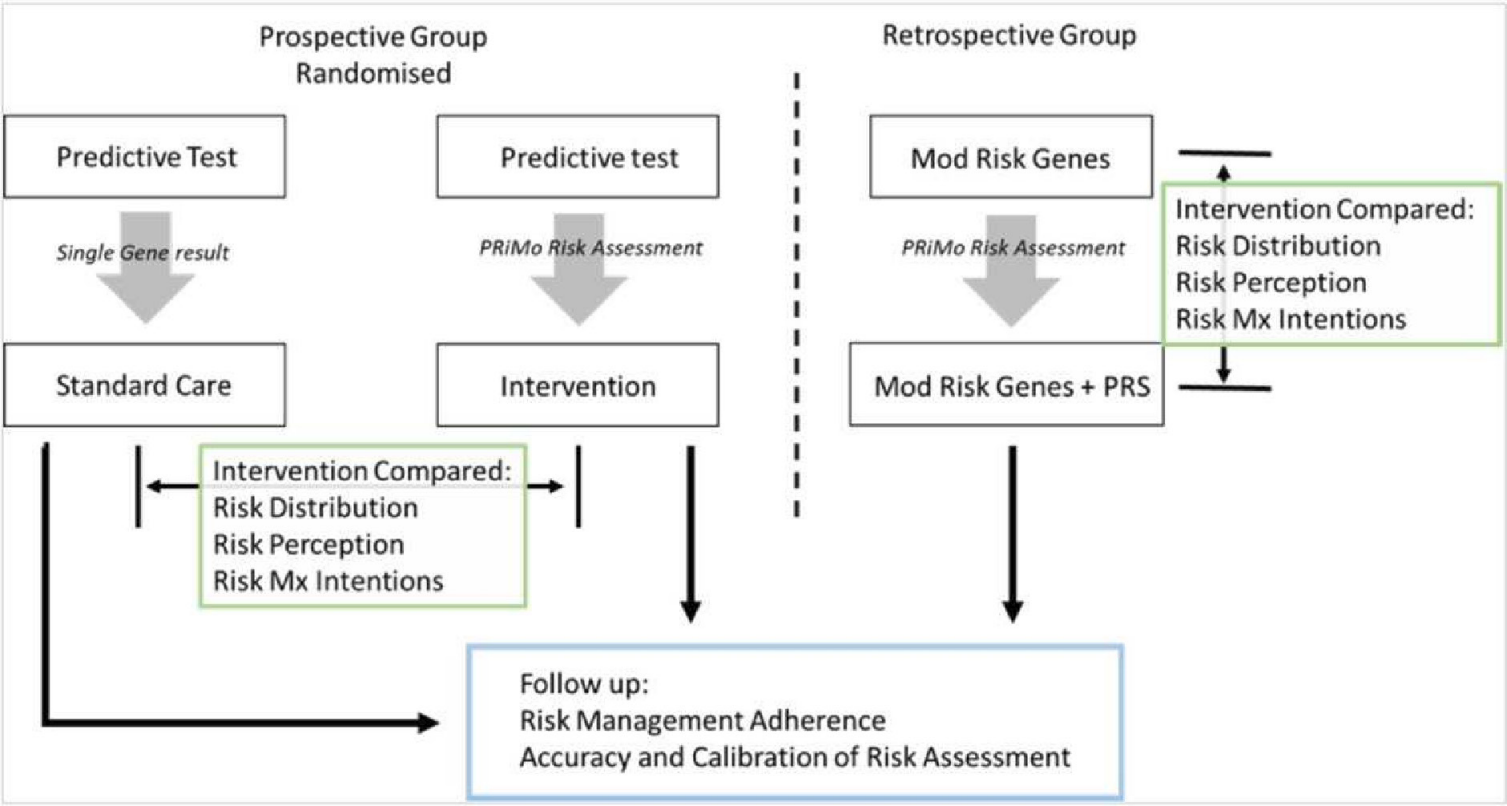
Outline of intervention and standard care group comparisons to assess short-term outcomes for the prospective RCT and the retrospective cohort. Mod Risk, moderate risk; Risk Mx, risk management. RCT, randomised controlled trial.

Figure 3 shows the between-group (RCT) and within-group (retrospective cohort) comparisons for the outcome measures.

#### Quantitative analysis

Demographic and other cohort features will be examined by descriptive statistics and include tests for heterogeneity between study sites and laboratories. Cohort variables will be compared between the study group and study decliners and with participants who withdraw or do not complete the prescribed assessments at each time point, as well as between the intervention groups.

Risk management and clinical endpoints: Proportions will be examined as above and rates (screening episodes, risk-reducing measures, cancer events) will be analysed by Cox regression and associations between variables and outcomes analysed by regression modelling. To account for multilevel clustering in the data (family, study site, gene, mutation type), a linear mixed model incorporating fixed and random effects corresponding to each level of hierarchy in the data or generalised estimating equation will be used. Predictive accuracy and calibration will be measured by the Matthews Correlation Co-efficient and calibration by a χ^2^ goodness of fit.

Participant experience: Internal consistency of the psychosocial tools will be measured by Cronbach’s alpha coefficient. The impact of variables, including the PRS, on psychosocial outcomes will be analysed in a linear mixed model incorporating data on other stressful life events and accounting for clustering as above.

#### Qualitative analysis

Interviews and focus groups will be transcribed and thematic analysis performed to examine perceived benefits and obstacles to testing perceived priorities, determinants for future clinical implementation, and the strategies, tools or resources that may be required. Initial results may inform the content of questionnaires at later study timepoints.

#### Microsimulation of long-term outcomes and healthcare implications

Primary outcome data from the follow-up and questionnaire data will be used to perform simulation of long-term (lifetime) outcomes to examine the practice implications for the Australian public healthcare system. The analysis uses miBRovaCAre, a complex model (a discrete-time, state-based microsimulation, with annual cycles) of high-risk management for breast and ovarian cancer^39^ that has been validated against prospective outcomes for the management of carriers of *BRCA1* and *BRCA2* PVs.^14^

#### Cost-effectiveness analysis

The longitudinal quality-of-life scores from the AQoL-8D (validated in an Australian setting and responsive to psychosocial measures^40 41^) will be used with ‘cost of care’ data gathered from linkage to the MBS, the PBS, hospital costs from government data and the main study outcomes, to evaluate the most effective and cost-effective care pathways for women at different levels of current and future breast cancer risk. The outputs include life-years and quality-adjusted life-years saved, healthcare costs associated with the intervention and the incremental cost-effectiveness ratio. The impact of uncertainty on model outputs will be assessed using one-way and probabilistic sensitivity analyses.

### Patient and public involvement

Collaboration with consumers and community members has been integral to the design of the study. Two participants in the pilot project of PRS-based assessment have acted as consumer advisors for the development of the trial and the final research plan. Consumer input was sought in the design of patient study materials and we have engaged a Consumer Advisory Panel to provide advice on the trial and dissemination of the study findings.

### Data management, monitoring and access

Study data will be collected and managed using the REDCap electronic data capture tools hosted at Peter MacCallum Cancer Centre. To promote data quality and participant retention, data collection forms include a range of checks and branching logic, and participants will be allowed to skip questions. Automated email and SMS reminders will be used, and participants will be invited to complete questionnaires at all timepoints, regardless of whether previous questionnaire(s) have been completed.

On written request, the full protocol, metadata and deidentified study data (excluding data provided by Services Australia), from participants who give permission for their data to be used for related future research projects can be made available to research collaborators contingent on approval by a Human Research Ethics Committee and the trial steering committee.

A trial steering committee meets 2 monthly to oversee study planning and progress and review adverse events and unintended consequences reported by site investigators to the central study site. Given the low-risk intervention, there will not be an additional independent Data Monitoring Committee.

## Ethics and dissemination

Ethics approval was granted by the Peter MacCallum Cancer Centre Human Research Ethics Committee (Melbourne, Victoria, Australia) in November 2020 (HREC/64060/PMCC). Research governance authorisation at each site was established prior to recruitment initiation. The trial is sponsored, and may be audited, by the Peter MacCallum Cancer Centre. Protocol modifications are approved by the ethics committee and sponsor (current approved protocol is v3.4 dated 8 November 2023). The sponsor and funder have no direct involvement in study design, data collection, analysis or decisions to publish the data. All participants are required to provide informed consent and individuals will not be identifiable in study reports or publications. Enrolment began in June 2021 and is expected to end in January 2025. Study participants may receive brief updates on the trial progress with their annual questionnaires and will receive an electronic summary of the study findings once the trial is completed. The trial is registered on the Australian New Zealand Clinical Trials Registry ACTRN12621000009819.

The trial data and implementation aims of the study are intended to be aligned with similar programmes internationally.^42^ The results of the PRiMo trial will be disseminated through peer-reviewed publications and presentation to the scientific community, and contribute to the international effort to understand how best to use this information to improve patient care.

## supplementary material

10.1136/bmjopen-2024-087874 online supplemental file 1
