# Supplementary material for: Using polygenic risk modification to improve breast cancer prevention: study protocol for the PRiMo multicentre randomised controlled trial
Source: BMJ Open. 2024 Aug 5;14(8):e087874. doi: 10.1136/bmjopen-2024-087874 (PMC11308879; doi:10.1136/bmjopen-2024-087874)
Supplement: online supplemental file 1 [file bmjopen-14-8-s001.pdf]

Participant Information Sheet

**Short Title**	The PRiMo Trial
**Title**	Using Polygenic Risk Modification to improve breast cancer prevention
**Coordinating Principal Investigator**	Prof Paul James
**Project Sponsor**	Peter MacCallum Cancer Centre
**Site Principal Investigator**	
**Location**	

1 Introduction

You are invited to take part in this research project which is called 'The PRiMo Trial'. This is because either:

i) a member of your family has been shown to carry an alteration (mutation) in one of the breast (or ovarian) cancer susceptibility genes (*BRCA1, BRCA2, PALB2, CHEK2, ATM, RAD51C* or *RAD51D*) and you have arranged an appointment with a Family Cancer Clinic (Genetics Service) to discuss having a genetic test to see if you carry this same alteration, or

ii) you have already had this genetic testing and have been found to carry a genetic alteration.

This research project is testing a new way of assessing a person's risk of developing cancer. It is called a 'personalised risk assessment' and includes a new type of genetic testing called genomic or 'Polygenic' testing.

This Participant Information Sheet/Consent Form tells you about the research project. It explains what participants will be asked to do as part of the project. Knowing what is involved will help you decide if you want to take part in the research.

Please read this information carefully. Ask questions about anything that you don't understand or want to know more about. Before deciding whether or not to take part, you might want to talk about it with a relative, friend or your local doctor.

If you decide you want to take part in the research project, you will be asked to electronically sign the consent form. By signing it you are telling us that you:

- Understand what you have read
- Consent to take part in the research project
- Consent to participate in the study procedures that are described below
- Consent to the use of your personal and health information as described.

You will be able to save a copy of this Participant Information Sheet and Consent Form.

Your participation is voluntary

Participation in this research is completely voluntary and there will be no cost to you. If you do not want to take part in this research project you do not have to. You should feel under no obligation to participate in this research. Choosing not to take part in this research will not affect your current and future medical care in any way.

Your withdrawal from the study

You are under no obligation to continue with the research project. You may change your mind at any time about participating in the research. People withdraw from research for various reasons and you do not need to provide a reason. You can withdraw from this research at any time by notifying a member of the research team.

2 What is genetic research?

Genes are made of DNA – the chemical structure carrying your genetic information that determines many human characteristics such as the colour of your eyes or hair.

Researchers study genes in order to try to understand why some people develop cancer and why some people do not. Understanding the information in a person's genes may help to work out who may benefit most from different strategies designed to prevent cancer.

3 What is the purpose of this research?

When someone has been shown to carry an alteration (mutation) in a breast (or ovarian) cancer susceptibility gene (*BRCA1, BRCA2, CHEK2, ATM, PALB2, RAD51C, RAD51D*), their family members can have an appointment with a Family Cancer Clinic to organise a genetic test to find out if they have or have not also inherited the alteration. This is sometimes called 'single gene' testing. This test result, along with family history information, can help genetic specialists assess the chance that someone will develop cancer (their level of risk). For individuals at higher risk, the genetic specialist will recommend effective ways of managing this risk. This type of testing and follow up is currently provided by genetics services across Australia, and is considered to be standard practice.

Technological advances in genetic testing have identified variants (small genetic changes) that are common but may make an important contribution to the risk of cancer for some people when testing for all of these variants is combined. This new testing is known as genomic or polygenic testing. Research has also identified other risk factors, such as use of particular hormonal medications, body mass index, and alcohol consumption that can help us to better predict the likelihood that a person will develop cancer.

The purpose of this research is to test a new 'personalised' way of assessing a woman's risk of developing breast and ovarian cancer. This personalised assessment will combine 'single gene' testing and family history information with genomic testing (the PRS) and additional risk factors. The research will determine if this can prevent cancers by improving our ability to determine the most appropriate risk management strategies in women who are undergoing (or have already had) genetic testing for an alteration (mutation) in a breast (or ovarian) cancer susceptibility gene. We will also assess women's experiences and feelings, and the health economic implications of this new approach to help us understand how this might become part of clinical practice in the future. The personalised risk assessment being tested in this study is accurate for individuals who are assigned female sex at birth. For this reason, the terms "women" and "woman" in this study are limited in their meaning, referring only to individuals who are assigned female sex at birth.

4 What does participation in this research involve?

There are seven main areas of participation that we ask you to consider:

a. Personalised risk assessment

You will be participating in a randomised controlled research project. We do not know if this new 'personalised risk assessment' that includes genomic testing is better than current standard practice. To find out, we need to compare it to the way the clinics currently perform a risk assessment. To do this we put participants into two groups. One group will receive the usual clinical risk assessment including the results of their single gene testing. The other group will receive the more personalised risk assessment including the results of their single gene testing and genomic/polygenic testing. To try to make sure the groups are the same, each participant is put into a group by chance (random). After you've completed your first research questionnaire, which will be before your appointment to receive your results, we will email to

tell you if you have been randomly assigned to receive standard results or the personalised risk assessment including genomic testing. This research project is also a cross-over study. This means that if you are in the group that receives the usual clinical risk assessment you will be asked one year after receiving your genetic test results if you would like to receive the personalised risk assessment and genomic testing results.

This research project has been designed to make sure the researchers interpret the results in a fair and appropriate way and avoids study doctors or participants jumping to conclusions.

If you have previously had testing and been shown to carry a genetic alteration you will not be randomised; you will be put in the group that will receive the personalised risk assessment and genomic testing results.

b. Online questionnaires

You will be asked to complete up to seven online questionnaires over a period of four years.

The first questionnaire will be emailed to you after your first appointment at the clinic to discuss genetic testing. If you have already had genetic testing, it will be emailed to you following a phone call to confirm your willingness to participate. The first questionnaire must be completed within 2 weeks because your responses may be required for your results appointment. If you do not complete this first questionnaire we cannot complete the research risk assessment and so you will be automatically withdrawn from the research.

Questionnaires will then be emailed to you approximately 4 weeks, 6 months, 1 year, 2 years and 3 years after you receive your genetic testing results. If you are in the group that receives the usual clinical risk assessment and you decide one year after receiving your genetic test results that you would like to receive the personalised risk assessment and genomic testing results we will ask you to complete an additional questionnaire.

The questionnaires will take 10 to 30 minutes to complete. The questionnaires will be completed by participants in both the standard clinical care and personalised risk assessment groups. You will be asked questions about your medical history that relate to cancer and cancer prevention strategies, and about lifestyle and other cancer risk factors. You will also be asked questions about your experience and feelings receiving your results.

Following review of your questionnaire responses you may be contacted by the researchers to clarify the information you have provided, or to offer you additional information or support.

c. Access to clinical information

We request your permission to access and collect your clinical information during the research project. This may include your medical history of cancer, demographics, genetic testing results and the details of screening tests, procedures, surgeries, use of medications and clinical advice relating to cancer treatment, prevention or risk assessment. So that we can perform the risk assessment, we will also collect your family tree with your family members' cancer history and genetic testing status (the family tree collected from your clinic record will have the names of your family members removed).

To collect this information we will access your Family Cancer Clinic medical record. We may also request this information from other hospitals, health services, medical specialists, pathology laboratories and medical or population registries including the state based cancer registries and the National Death Index.

We may provide information that we have collected from these other sources and your questionnaires to your Family Cancer Clinic so that it is available for your ongoing clinical care.

d. Access to Medicare Benefits Schedule (MBS) and Pharmaceutical Benefits Scheme (PBS) information (optional)

You will be asked to sign a separate consent form authorising the study to access your complete Medicare Benefits Schedule (MBS) and/or Pharmaceutical Benefits Scheme (PBS) data as outlined in the consent form. Medicare collects information on your doctor visits and the associated costs, while the PBS collects information on the prescription medications you have filled at pharmacies. Giving permission for the release of your claims information is optional and you do not have to provide this permission to participate. We will provide you with

this MBS/PBS consent form via email. If you provide your permission your consent form will be sent securely to Services Australia who holds MBS and PBS data confidentially.

You can withdraw your consent for MBS/PBS data access at any time by completing and signing the 'Participant Withdrawal of Consent form' that is provided at the end of the separate MBS/PBS consent form or request this by contacting the study team. This form is to be completed by you and supplied to the research team at a later date. If you decide to withdraw your consent for researchers to access your MBS/PBS data, you will be able to choose whether the study will destroy or retain the information it has collected about you from Services Australia. You should only choose one of these options on the form. Where both boxes are ticked in error or neither box is ticked, the study will destroy all information it has collected about you. If you choose to withdraw your consent for MBS/PBS data collection you do not have to withdraw from the whole research project if you do not want to.

e. Access to biological samples

(i) DNA sample

Your genetic specialist will organise for you to have a DNA (blood) sample collected to test for the alteration (mutation) in your family. If you decide to participate in this research project, we will ask for some of this DNA sample to be sent to our research laboratory.

If you have already had testing for the alteration in your family we will ask the laboratory that performed the testing to send some of your stored DNA sample to our research laboratory.

This research sample will be used for genomic testing to look for common and rare known genetic variations that are associated with breast or ovarian cancer risk.

(ii) Diagnostic Tissue Block and/or DNA sample

If you were to develop breast or ovarian disease during the research project we request your permission to collect any tissue left over from biopsies or surgery, and to collect any tissue that remains from breast or ovarian surgery you might have had in the past. This will be done in only some cases, particularly where the tumour sample is required to study the characteristics of the cancer and the association with polygenic risk. We will only use left over tissue after a clinical diagnoses is made and treatment is planned.

f. Interviews (optional)

A part of this research project will include a telephone or in-person interview for some participants. This will only include a group of 30 to 60 participants out of the several hundred that will be involved in the research project. If you choose to be involved (and are selected), the interviews will be conducted around 1-4 months and approximately 12 months after you receive your genetic testing results. Interviews will take around 30-60 minutes. You will be asked questions about your experience and feelings receiving your results. With your permission, the interview will be audio taped. If you wish to stop the interview you may do so at any time. Following the interview the audiotapes will be transcribed verbatim (word for word) by a professional transcription service with experience transcribing research interviews, such as Transcriber Online. Your interview transcript and audio recording will be stored for the duration of the study and required retention period in an access restricted electronic folder on a server at Peter MacCallum Cancer Centre. The copy of the audio file transferred securely to the transcription service will be deleted by them within 60 days of the transcription being completed.

g. Focus groups (optional)

Another part of this research project will include virtual or in-person focus groups for some participants. Each focus group will include 6-10 individuals. If you are interested in being part of a focus group (and are selected), the focus groups will occur from around 12 months after the commencement of enrolment, and will primarily involve participants randomised to the standard-care arm and are considering cross-over to receive their personalised risk assessment. Focus groups are expected to take 1-2 hours to complete. You will be asked questions about the acceptability and value of the personalised risk assessment and how you think it should be delivered to patients in the real-world clinical setting. With your permission, the focus group will be audio taped. Following the focus group, the audiotape will be transcribed verbatim (word for word) by a professional transcription service, such as

Transcriber Online. The focus group transcript and audio recording will be stored for the duration of the study and required retention period in an access restricted electronic folder on a server at Peter MacCallum Cancer Centre. The copy of the audio file transferred securely to the transcription service will be deleted by them within 60 days of the transcription being completed.

5 What do I have to do?

The research results and personalised risk assessment will be provided during your Family Cancer Clinic appointment. If you are randomly assigned to receive your single gene test result, and you decide after one year that you would like to receive your 'personalised risk assessment' and genomic testing result, we will organise an appointment with your Family Cancer Clinic at that time.

If you have already had genetic testing and been shown to carry an alteration we will organise for you to have an additional appointment with your Family Cancer Clinic to receive your research results.

You will also be asked to:

- Read this Participant Information Sheet
- Enter your details and answer the screening questions
- Sign the online consent form
- Complete up to seven online questionnaires over a period of four years
- Sign the MBS/PBS consent form (optional)
- Participate in an interview (optional and only 30 to 60 participants)

6 Other relevant information about the research project

We aim to recruit a large number of women (approximately 2400) through the Family Cancer Clinics across Australia.

There are no costs associated with participating in this research, nor will you be paid. The research testing and any appointments that are related to the research project will be provided to you free of charge.

The research testing of your DNA sample will be carried out at one of the participating laboratories located at:

- Precision Medicine, School of Clinical Sciences at Monash Health, Melbourne
- Australian Translational Genomics Centre, Institute of Health and Biomedical Innovation, Queensland University of Technology, Brisbane
- Molecular Diagnostic Laboratory, Peter MacCallum Cancer Centre, Melbourne

7 Do I have to take part in this research project?

Participation in any research project is voluntary. If you do not wish to take part, you do not have to. If you decide to take part and later change your mind, you are free to withdraw from the project at any stage. If you decide to withdraw, please notify a member of the research team. You will be provided with a 'Form for Withdrawal of Participation' to complete and sign.

From the withdrawal date researchers will not collect additional information from or about you, although information already collected will be retained to ensure that the results of the research project can be measured properly and to comply with law. If you withdraw from the research your biological samples collected for diagnostic testing, including any DNA collected to test for the alteration in your family, will continue to be stored by the diagnostic laboratory but any sample(s) sent to the research laboratory will be discarded.

If, at the time that you withdraw, researchers have collected your MBS/PBS information from Services Australia, you will be asked to complete and sign an additional withdrawal form to tell the researchers how you want them to handle your MBS/PBS information.

Your decision whether to take part or not to take part, or to take part and then withdraw, will not affect your routine care or your relationship with the Family Cancer Clinic.

The first online questionnaire allows us to gather information that is relevant to the personalised risk assessment and is required for your results appointment. Therefore, if you

are unable to complete this first questionnaire we will have to automatically withdraw you from the research project. This will not affect your clinical care and you will still be able to attend your clinic appointment to receive your single gene genetic test result.

During your first appointment at the Family Cancer Clinic there will be an opportunity to discuss both the clinical genetic testing and this research with your clinician. If at that appointment you decide that you don't want to go ahead with the clinical genetic testing we will also organise to withdraw you from the research project. Again, this would not affect your continuing care through the Family Cancer Clinic.

8 What are the possible benefits of taking part?

We cannot guarantee or promise that you will receive any benefits from this research. However, participation will provide you with the opportunity to receive a personalised risk assessment including genomic testing. This is not the current standard of care in the Family Cancer Clinics and is offered as a research test. Through your participation you will provide information that will be used to determine if this new approach is better than the current standard of care, and may inform how best to implement this approach into future clinical practice.

9 What are the possible risks and disadvantages of taking part?

A personalised risk assessment that includes genomic testing may raise important issues. In some cases, the result could create uncertainty or be upsetting; if for instance they indicate an increased risk for breast and/or ovarian cancer. If you become upset or distressed as a result of your participation in this research project, the research team will arrange for counselling or other appropriate support. Any counselling or support will be provided by qualified staff members who are not members of the research team. This counselling will be provided free of charge.

Statutory or contractual duties may require you to disclose results of genetic tests or analysis to third parties (for example, insurance companies, employers, financial and educational institutions), particularly where results provide information about health prospects.

If you decide to obtain the results of your genetic tests, you may then be obliged to disclose this on any future application for insurance cover over specified limits or for certain types of employment should it be requested. This is true both for the clinical genetic test and the research genetic testing however the risk of insurance implications are higher for the single gene testing that you will be offered as part of standard care by your Family Cancer Clinic.

Currently in Australia there is an industry-based moratorium on the use of genetic information in the assessment of life or income insurance up to a certain value. Under this arrangement you are able to access up to a specified level of life or income cover without disclosing a genetic test result or information about your participation in genomic research to your insurer.

10 What will happen to my test samples?

A small sample of your DNA collected to test for the alteration in your family will be sent to a research laboratory associated with this research project for genomic testing. If you were to develop breast or ovarian disease in the future, we may collect a sample of any tissue left over from biopsies or surgery. The remainder of your DNA and any tissue sample will be retained by the diagnostic laboratories originally responsible for them.

The research DNA and tissue samples will be retained in the research laboratories for the duration of the research project or associated continuing research. We would like to store the research samples for future use in research projects that are closely related to this research project. Such research would have to comply with the permissions described in this information sheet and would only occur with specific approval from an ethics committee.

11 Will I be given the results of the research project?

In this research project all participants have the opportunity to receive the results of additional personal genomic testing – assessing the delivery and value of this information is the main research project activity. In addition, all participants will receive a summary of the main results of the research when these become available at the end of the research project.

Our genes contain information that is relevant to many different health problems. In this research project we will be focusing only on genes that contribute to cancer risk and we will not analyse genes for other conditions. As a result, the risk that we would uncover information about unrelated health problems is low.

However, if during the course of this research project an unintended finding of this type occurs that may be important for your (or your family's) health care, it will be carefully examined by an appropriate team of ethical and medical advisors. If such a situation was to arise we may contact you so that you can decide if you want to learn more about that information.

12 What will happen to information about me?

By signing the consent form you consent to the research team collecting and using personal information about you for the research project. Any information obtained in connection with this research project that can identify you will remain confidential. It will only be disclosed with your permission, except as required by law.

Information about you may be obtained from your health records held at this and other health services for the purpose of this research. By signing the consent form you agree to the research team accessing health records if they are relevant to your participation in this research project.

Information you provide to the research team or give us permission to collect will be stored electronically in a secure database or restricted electronic folders housed on secure servers at the Peter MacCallum Cancer Centre. Your information will be stored in such a way that your identity could reasonably be ascertained. Only those persons authorised will have access to the information.

If you provide permission for us to access your MBS and PBS claims information, the information and your electronically signed consent form will be stored electronically in a secure database or restricted electronic folders, physically located on servers within Australian borders.

When your information is analysed, all identifiers (e.g. name and personal details) will be removed and replaced with a code. It will be possible to re-identify the information as yours using the code.

Information about your participation in this research project may be recorded in your health records at the Family Cancer Clinic. Information that we collect from other health services and your questionnaires may also be recorded in your Family Cancer Clinic record so that it is available for your ongoing clinical care.

Processing of your genomic data will involve use of external online computational tools (including tools hosted internationally). No identifying data will be shared with the external entities managing and hosting the online computational tools and no data will be retained by these external entities following the data analysis process.

With your consent we would like to store your information for future use in research projects that are closely related to this research project. Such research would have to comply with the permissions described in this information sheet and would only occur with specific approval from an ethics committee. Future research projects would not include your MBS/PBS claims data – this data will not be used for any research outside of this approved project.

In accordance with the institutional policy of Peter MacCallum Cancer Centre where this research project is being conducted, your study records and data will be retained for a minimum of 15 years from the date of completion of all research activities. After the completion of the study and at the end of the retention period, your confidential information that has been stored electronically will be destroyed securely using a specialized 'shredding' software and any hard copy documents will be shredded.

Your health records and any information collected and stored by the research team during the research project may be reviewed for the purpose of verifying the procedures and the data. This review may be done by the ethics committee which approved this research project, regulatory authorities and authorised representatives of the Sponsor, Peter MacCallum Cancer Centre or as required by law. In these circumstances, the Sponsor will not collect your

personal information. By signing the consent form, you authorise release of, or access to, this confidential information as described above.

According to the Australian and state privacy and other relevant laws, you have the right to request access to your information collected and stored by the research team. You also have the right to request that any information with which you disagree be corrected. Please contact the research team named at the end of this document if you would like to access your information.

Results from this research project will be published in scientific or medical journals and presented at scientific meetings and potentially in the wider media. For these reports no identifying information will be included, and care will be taken to ensure that it is not possible to identify you from public reports, except where you have given your express permission.

13 Who is organising and funding the research?

This research project has been initiated by the study doctor, Professor Paul James and is conducted by the Parkville Familial Cancer Centre, Peter MacCallum Cancer Centre, in collaboration with Family Cancer Clinics and Genetics Services across Australia.

This research project has been funded by the National Breast Cancer Foundation and Love Your Sister Foundation, Australia.

No member of the research team will receive a personal financial benefit from your involvement in this research project (other than through their regular employment).

14 Who has reviewed the research project?

All research in Australia involving humans is reviewed by an independent group called a Human Research Ethics Committee (HREC). The ethical aspects of this research project have been approved by the HREC of Peter MacCallum Cancer Centre.

This project will be carried out according to the *National Statement on Ethical Conduct in Human Research (2007).* This statement has been developed to protect the interest of people who agree to participate in human research studies.

15 Further information and who to contact

The person you may need to contact will depend on the nature of your query.

If you want any further information concerning this project or if you have any problems which may be related to your involvement in the project, you can contact the site principal investigator on xxxxxxxxx or any of the following people:

Study contact person

Position	
Telephone	
Email	

For matters relating to research at the site at which you are participating, the details of the local site complaints person are:

Complaints contact person

Name	
Position	
Telephone	
Email	

If you have any complaints about any aspect of the project, the way it is being conducted or any questions about being a research participant in general, then you may contact:

Reviewing HREC approving this research and HREC Executive Officer details

Reviewing HREC name	
HREC Executive Officer	

Telephone	
Email	

Local HREC Office contact

Name	
Position	
Telephone	
Email	

If you have a privacy complaint in relation to the use of your MBS/PBS data you should contact the Office of the Australian Information Commissioner. You will be able to lodge a complaint with them.

Website	
Telephone	
Email	
Mail	

Consent Form

**Short Title**	The PRiMo Trial
**Title**	Using Polygenic Risk Modification to improve breast cancer prevention
**Coordinating Principal Investigator**	Prof Paul James
**Project Sponsor**	Peter MacCallum Cancer Centre
**Site Principal Investigator**	
**Location**	

Consent Agreement

I have read the Participant Information Sheet.

I understand the purposes, procedures and risks of the research described in the project.

I have had the opportunity to ask questions.

I freely agree to participate in this research project as described and understand that I am free to withdraw at any time during the project without affecting my future health care.

I understand that I may keep an electronically signed copy of this document.

I give permission for my Family Cancer Clinic, and doctors, other health professionals, hospitals, registries, other health services or laboratories outside this hospital, to release information, stored DNA and tissue samples to the Peter MacCallum Cancer Centre that are needed for this project. I understand that such information will remain confidential.

I give permission for the use of my samples and information collected for this research project for the purpose of *(must select one option)*

- ❑ this research project and a direct extension of this project only, or
- ❑ this research project and a direct extension of this project, and other closely related future research projects that have been approved by a Human Research Ethics Committee.

This research project includes interviews and focus groups, via telephone, videoconference or in-person, with a sub-set of participants. You can opt-out of the telephone, videoconference or in-person interviews and focus groups by ticking the box below.

- ❑ I wish to participate in this research project, but please **do not** contact me for an interview or focus group.

Name of participant
Electronic signature
Date
