# Supplementary material for: Using polygenic risk modification to improve breast cancer prevention: study protocol for the PRiMo multicentre randomised controlled trial
Source: BMJ Open. 2024 Aug 5;14(8):e087874. doi: 10.1136/bmjopen-2024-087874 (PMC11308879; doi:10.1136/bmjopen-2024-087874)
Supplement: Supplementary file 2 [file bmjopen-2024-087874.reviewer_comments.pdf]

PEER REVIEW HISTORY

BMJ Open publishes all reviews undertaken for accepted manuscripts. Reviewers are asked to complete a checklist review form (**http://bmjopen.bmj.com/site/about/resources/checklist.pdf**) and are provided with free text boxes to elaborate on their assessment. These free text comments are reproduced below.

ARTICLE DETAILS

**TITLE (PROVISIONAL)**	Using Polygenic Risk Modification to improve breast cancer prevention: study protocol for the PRiMo multicentre randomised controlled trial
**AUTHORS**	McInerny, Simone; Mascarenhas, Lyon; Yanes, Tatiane; Petelin, Lara; Chenevix-Trench, Georgia; Southey, Melissa C.; Young, Mary-Anne; James, Paul

VERSION 1 – REVIEW

**REVIEWER**	Imoto,, Issei<br>Aichi Cancer Center Research Institute
**REVIEW RETURNED**	05-May-2024

**GENERAL COMMENTS**	This is an excellent protocol paper and I have no serious criticisms regarding methodology to perform this study.

**REVIEWER**	Lambert, , Samuel A<br>University of Cambridge
**REVIEW RETURNED**	24-May-2024

**GENERAL COMMENTS**	McInerny et al. describes an interesting RCT evaluating the impact of including PRS into intergrated cancer risk assesments in the Australian clinical setting. The study is well described but I have some questions where additional context would be helpful:<br><br>It would be good to further explain why such short terms risks being predicted (5/10-year)? What is the average expected age of participants, would lifetime risk be more informative for younger individuals? What is the format of the risk assessment report, could an example be added to the protocol?<br><br>The information about how the genetic testing and PRS is underspecified. Some questions:<br>• Is there a standard reference/protocol for how PVs are being identified? How will VUS carriers be handled within the trial?<br>• It would be useful to know which imputation server/panel and software are being used – will these panels be customized or matched by participant ancestry?<br>• What software will be used to calculate and convert the PRS SUM into a Z-score?<br>o Will any adjustments be made to decorrelate the PRS from genetic ancestry (e.g. methods that adjust PRS using genetic PCs, Linder et al. (2023) Genet Med; Hao et al. (2022) Nature Med)?<br>o How will you decide which populations mean and standard deviation to apply to the raw PGS to convert it into a Z-score? Is

	this by self-reported ethnicity or will you determine genetic ancestry?<br><br>Pertaining to the psychosocial data:<br>• Will participants be offered interviews randomly? How will responder bias be captured in these results?<br>• There are quite a few questionaries being surveyed, was the number of surveys deemed acceptable to participants in the pilot trial or co-design?<br><br>Pertaining to the power calculations:<br>• What is the event rate you expect within the trial? The power is written in-terms of proportion of cases; however, this must also require some assumption about how many cases will be observed?<br>• What does “more than adequate power” correspond to? How will multiple-testing burden be accounted for with so many tests.

VERSION 1 – AUTHOR RESPONSE

Reviewer: 2

1. It would be good to further explain why such short terms risks being predicted (5/10-year)? What is the average expected age of participants, would lifetime risk be more informative for younger individuals? What is the format of the risk assessment report, could an example be added to the protocol?

Thank you for this comment. Participants are provided with 5-year, 10-year and lifetime risks – we should have made this clearer in the manuscript. We agree that lifetime risk is often the most important risk figure, particularly for younger women.

We have updated the sentence:

This personalised risk assessment is calculated using the CanRisk tool and the output summarised in a custom report that includes the primary genetic information and a risk summary of 5-, and 10-year <u>and lifetime</u> risks for breast and ovarian cancers.

We have now included an example of the front summary page of the reports in the supplementary material.

2. The information about how the genetic testing and PRS is underspecified. Some questions:
• Is there a standard reference/protocol for how PVs are being identified? How will VUS carriers be handled within the trial?

All testing for pathogenic variants in the study is performed in clinically accredited diagnostic laboratories and the study itself is not involved in the variant curation for the standard genetic testing element. The tests being performed are all predictive – that is the familial variants being tested for have all been previously classified as pathogenic/likely pathogenic by a diagnostic laboratory through testing for another family member. Predictive testing is not offered for VUS in clinical practice and as the testing is focused just on the familial variant these won’t arise in this study.

We have updated the sentence:

Study participants will be recruited through 11 dedicated FCCs around Australia where unaffected women are referred for predictive genetic testing of high- or moderate-risk breast or ovarian cancer genes, following detection of a <u>likely pathogenic (class 4) or</u> pathogenic <u>(class 5)</u> variant in a family member.

• It would be useful to know which imputation server/panel and software are being used – will these panels be customized or matched by participant ancestry?
We have added the information on the datasets used for imputation. Currently the panels are not matched by ancestry, however it is worth noting that more than 90% of the SNPs are genotyped directly on the array so that the fraction of imputed data is small (in contrast to a GWAS study for instance). A sensitivity analysis comparing the centile of PRS for the first ~500 individuals genotyped with or without the imputed SNPs indicated that there was minimal impact on classification.
We have updated the sentence:
Missing genotypes, either due to limitations of the array or issues arising from individual samples, are managed by imputation against the <u>TOPMED and 1000 Genomes</u> whole-genome data sets using standard methodology <u>to optimise the imputation accuracy for each SNP</u>.

• What software will be used to calculate and convert the PRS SUM into a Z-score?
We use our own custom tool to convert the genotypes into a z-score and adjust for ancestry (as described below). However this isn't complex in any way – it simply converts the genotypes to the corresponding Log(OR), adds them up and standardises the resulting score against the relevant established distribution based on ancestry.

- **Will any adjustments be made to decorrelate the PRS from genetic ancestry (e.g. methods that adjust PRS using genetic PCs, Linder et al. (2023) Genet Med; Hao et al. (2022) Nature Med)?**
- **How will you decide which populations mean and standard deviation to apply to the raw PGS to convert it into a Z-score? Is this by self-reported ethnicity or will you determine genetic ancestry?**

Yes, adjustment is made for ancestry for the breast cancer PRS. Because the study is applying the PRS prospectively it is not possible to do this using distributions generated within the study itself (as in the publications mentioned). Fortunately, the 313-SNP BC PRS has been examined in large cohorts for a number of broad ancestral groups (referenced in the protocol – refs 25,35,36) and the published mean and SD for these groups is used for the adjustment. This has the expected effect of reducing the predictive strength of the PRS component of the risk assessment for all ancestries other than European. For some ancestral groups (e.g. Indigenous Australians, Pacific Islanders) there is no information on the properties of the PRS and a conservative approach is used to adjust to a 'baseline' equivalent to the group where the PRS is least predictive.
In this study self-identified ancestry is used (based on information about 4 grandparents) but the data gathered will allow this to be converted to an internal assessment of ancestry based on the genotyping in the future. For individuals enrolled to date 92% have self-identified as European, with the next largest group being individuals of Asian descent.
Currently there is no equivalent information about the performance of the OC PRS in different populations and this is not being adjusted in the study. This approach was felt to be reasonable as the OC PRS is both significantly weaker in its prediction of risk (than the BC PRS) and a smaller component of the overall risk model.

3. Pertaining to the psychosocial data:
• Will participants be offered interviews randomly? How will responder bias be captured in these results?
As only a small fraction of participants will be included in the interview group invitations are based on purposive sampling to reduce the effect of responder bias and ensure that there is a representative distribution of individuals included, based on age, genetic result and the final level of risk assessed. The size of the group (~60 individuals) is large for a qualitative study with the aim of capturing both recurrent themes that emerge but also the diversity of experience/views.

We have expanded the description of this in the text:
To achieve a representative sample participants will be purposefully recruited to reflect the range of age, genetic result and cancer risk.

• There are quite a few questionaries being surveyed, was the number of surveys deemed acceptable to participants in the pilot trial or co-design?
The trial has been designed, in collaboration with consumers, to integrate study activities with existing clinical processes, to make the study less intrusive in terms of patient experience and minimise the burden on participants. For example, study processes are integrated into standard clinical appointments, where possible data is collected from the participant's medical record rather than directly from the participant, and the research testing is performed on the diagnostic sample so that participation doesn't require any additional sample collection. The platform used for the participant surveys was designed to minimise the burden on participants - participants are allowed to skip questions, branching logic is used, and whilst the surveys at earlier timepoints take approximately 30minutes to complete, they get shorter over time with the year 2 and year 3 surveys taking <5minutes to complete.
The range of psychosocial/QoL instruments included does follow an approach previously used in a pilot study [Yanes et al. Genet Med. 2021 Dec;23(12):2316-2323] that had a good rate of survey completion (87%) at 12 months.

4. Pertaining to the power calculations:
• What is the event rate you expect within the trial? The power is written in-terms of proportion of cases; however, this must also require some assumption about how many cases will be observed?
We have improved the description of the 'events' being compared in the main power calculation:
A power calculation was performed based on the frequency of cancer risk management events in cases and controls – including uptake of recommended strategies and reduction in strategies no longer recommended based on the participants risk assessment.
Based on the expected ~50% positive rate of the predictive genetic test the rate of risk management recommendations and uptake (events) is assumed to be high i.e. at least 50%, as it is in clinical practice.
We have avoided using the term 'case' to avoid confusion when discussing prospective cancer events – all participants are unaffected at enrolment. As noted we used a detailed model including the expected distribution of annual risk in the cohort (based on the expected distribution of age, genetic test results and a background normal distribution of the PRS) to project the expected incident cancers. Importantly this study does not aim to measure any impact on this outcome but as described we measured the power to demonstrate better calibration of the comprehensive model over standard risk assessment. We have tried to define this outcome in simple language in the text as:
"a greater proportion of the breast cancers detected in follow-up will occur in the high-risk women in the intervention arm than in the standard care arm"

• What does "more than adequate power" correspond to? How will multiple-testing burden be accounted for with so many tests.
The psychosocial instruments being used are typically applied in much smaller studies and even with a modest retention rate the power calculation for each exceeds usual standards for a well powered study – we have added in a definition of $\beta>90\%$, $\alpha=0.01$ to the sentence to clarify. There are 8 psychosocial instruments and AQoL, suggesting a multiple-testing threshold of $\alpha=0.0055$, (which we will apply for any of the instruments that do show a significant difference), although it is worth noting that the anticipated outcome is that personalised testing will not significantly impact patient psychosocial wellbeing. Consequently, we will follow a conservative approach when assessing potential evidence to suggest this isn't the case. Consideration will be given to both statistical significance and what represents clinically meaningful differences when interpreting this data.

The amended sentence now reads:
For the secondary aims, the proposed study size correlates to more than adequate statistical power (β>90%, α=0.01) to detect differences in the questionnaire outcomes. Consideration will be given to both the statistical and clinical significance of any differences.

VERSION 2 – REVIEW

**REVIEWER**	Lambert, , Samuel A<br>University of Cambridge
**REVIEW RETURNED**	10-Jul-2024

**GENERAL COMMENTS**	The authors have adequately addressed my comments with their revised manuscript. I look forward to seeing the results of the trial.
